# Endoscopic full-thickness resection with local injection into tissue outside the capsule of a gastrointestinal stromal tumor

**DOI:** 10.1055/a-2471-7995

**Published:** 2024-12-03

**Authors:** Makoto Kobayashi, Tatsuma Nomura, Junki Toyoda, Yuto Ikadai, Tomohiro Sase, Tomonori Saito, Katsumi Mukai

**Affiliations:** 1 37036 Department of Gastroenterology, Yokkaichi Municipal Hospital, Yokkaichi, Japan; 2 Department of Gastroenterology, Suzuka General Hospital, Suzuka, Japan

Recently, the usefulness of endoscopic full-thickness resection (EFTR) of gastrointestinal stromal tumor (GIST) has been demonstrated 1 2 . However, the R0 resection rate of endoscopy alone is problematic. We previously reported a method for achieving R0 resection during endoscopic muscularis dissection of an intraluminal growth type GIST using local injection of a hyaluronic acid solution into the muscle layer to attach the muscle layer to the tumor side using a knife capable of delivering the fluid 3 . In this report, we describe the first case of local injection of hyaluronic acid solution into tissue outside the GIST capsule to achieve R0 resection.

The patient was a 72-year-old woman with a 13-mm GIST underneath an early gastric tumor in the lesser curvature of the stomach ( Fig. 1 , Video 1 ). We performed ESD for the early gastric cancer, followed by complete resection of the GIST using EFTR ( Fig. 2 ).

**Fig. 1:**
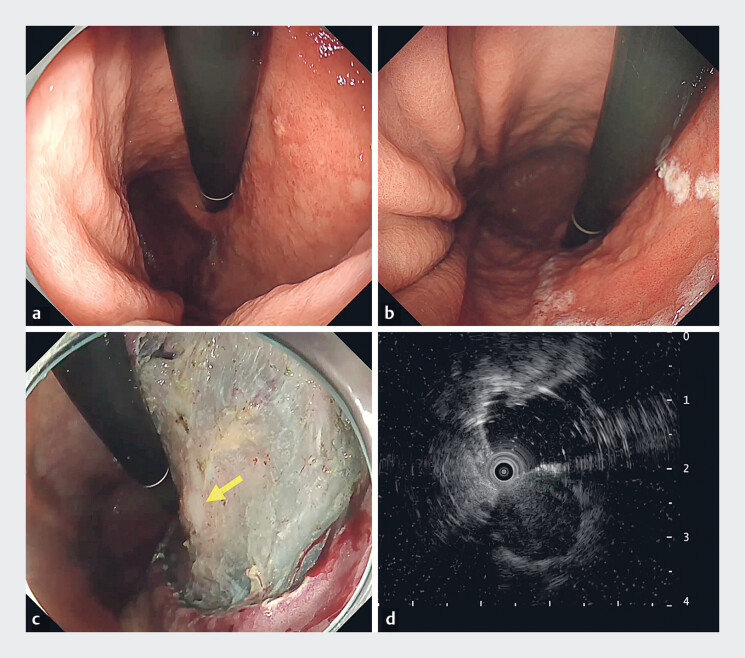
Endoscopic submucosal dissection (ESD) for early gastric cancer overlying a gastrointestinal stromal tumor (GIST). **a** Early gastric cancer lesion in the lesser curvature of the gastric body. **b** Early gastric cancer lesion after marking. **c** Post-ESD mucosal defect after resection of early gastric cancer. The underlying submucosal tumor is visible in the defect (yellow arrow). **d** Endoscopic ultrasound showed the GIST underneath the mucosal defect.

R0 resection achieved by endoscopic full-thickness resection with local injection into tissue away from a gastrointestinal stromal tumor.Video 1

**Fig. 2:**
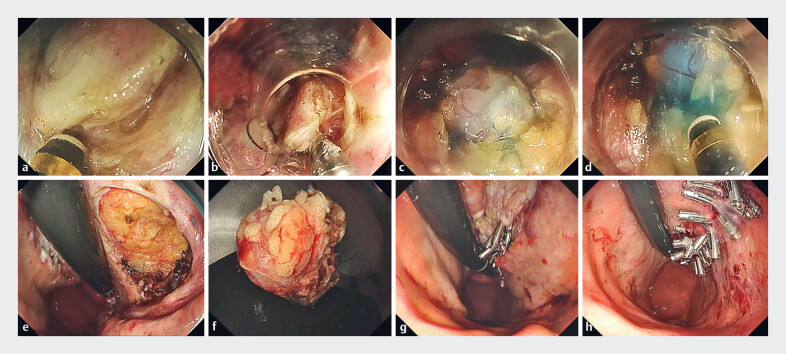
Endoscopic full-thickness resection (EFTR) using local injection into tissue outside the gastrointestinal stromal tumor (GIST) capsule. The GIST was located underneath the mucosal defect left by endoscopic submucosal dissection. **a** The muscle layer was incised away from the tumor. **b** A clip with a line was placed in the normal muscle layer on the anal side. **c** The submucosal tumor was exposed. **d** Local injection into tissue at an appropriate distance from the submucosal tumor to allow resection of tissue. **e** Full-thickness defect after EFTR. **f** Resected tumor specimen. **g** Site after closing the full-thickness defect using the reopenable clip over-the-line method (ROLM). **h** Full-thickness defect completely closed after a second ROLM.

The defect after ESD for early gastric cancer was 65 mm, and the GIST was located beneath the muscle layer of the mucosal defect. First, the muscle layer around the tumor was incised. A clip with line was placed on the anal side of the normal muscle layer. Local injection into the tissue outside the GIST capsule resulted in blue staining of tissue away from the tumor to allow precise and complete dissection of the GIST. Even fatty tissues that are difficult to resect can be removed easily by local injection as electrolytes are injected into the tissues.

The pathological diagnosis was R0 resection and a very low-risk GIST. The muscle layer of the full-thickness defect was closed using the reopenable clip over-the-line method (ROLM) 4 5 . The remaining mucosal defect was completely closed using ROLM. The patient was discharged 6 days after the procedure without any adverse events.

Endoscopy_UCTN_Code_TTT_1AO_2AG_3AF

## Notes

Endoscopy E-Videos https://eref.thieme.de/e-videos
*E-Videos* is an open access online section of the journal *Endoscopy* , reporting on interesting cases and new techniques in gastroenterological endoscopy. All papers include a high-quality video and are published with a Creative Commons CC-BY license. Endoscopy E-Videos qualify for HINARI discounts and waivers and eligibility is automatically checked during the submission process. We grant 100% waivers to articles whose corresponding authors are based in Group A countries and 50% waivers to those who are based in Group B countries as classified by Research4Life (see: https://www.research4life.org/access/eligibility/ ). This section has its own submission website at https://mc.manuscriptcentral.com/e-videos .
